# Loss of TrkB Signaling Due to Status Epilepticus Induces a proBDNF-Dependent Cell Death

**DOI:** 10.3389/fncel.2019.00004

**Published:** 2019-02-08

**Authors:** Laura Ester Montroull, Víctor Danelon, Andrea Beatriz Cragnolini, Daniel Hugo Mascó

**Affiliations:** Facultad de Ciencias Exactas, Físicas y Naturales, Universidad Nacional de Córdoba; Instituto de Investigaciones Biológicas y Tecnológicas (IIByT), CONICET-Universidad Nacional de Córdoba, Córdoba, Argentina

**Keywords:** matBDNF, proBDNF, TrkB, p75NTR, hippocampus, seizures

## Abstract

Neurotrophins (NTs) are secretory proteins that bind to target receptors and influence many cellular functions, such as cell survival and cell death in neurons. The mammalian NT brain-derived neurotrophic factor (matBDNF) is the C-terminal mature form released by cleavage from the proBDNF precursor. The binding of matBDNF to the tyrosine kinase receptor B (TrkB) activates different signaling cascades and leads to neuron survival and plasticity, while the interaction of proBDNF with the p75 NT receptor (p75NTR)/sortilin receptor complex has been highly involved in apoptosis. Many studies have demonstrated that prolonged seizures such as *status epilepticus* (SE) induce changes in the expression of NT, pro-NT, and their receptors. We have previously described that the blockage of both matBDNF and proBDNF signaling reduces neuronal death after SE *in vivo* (Unsain et al., 2008). We used an *in vitro* model as well as an *in vivo* model of SE to determine the specific role of TrkB and proBDNF signaling during neuronal cell death. We found that the matBDNF sequestering molecule TrkB-Fc induced an increase in neuronal death in both models of SE, and it also prevented a decrease in TrkB levels. Moreover, SE triggered the interaction between proBDNF and p75NTR, which was not altered by sequestering matBDNF. The intra-hippocampal administration of TrkB-Fc, combined with an antibody against proBDNF, prevented neuronal degeneration. In addition, we demonstrated that proBDNF binding to p75NTR exacerbates neuronal death when matBDNF signaling is impaired through TrkB. Our results indicated that both the mature and the precursor forms of BDNF may have opposite effects depending on the scenario in which they function and the signaling pathways they activate.

## Introduction

The neurotrophin (NT) brain-derived neurotrophic factor (matBDNF) plays an important role in neuronal differentiation and survival. matBDNF is initially synthesized as proBDNF and is released as a mature form through cleavage by furin or metalloproteinases (Seidah et al., 1996; Mizoguchi et al., 2011). On one hand, matBDNF deploys its biological functions *via* two transmembrane receptors: tropomyosin receptor kinase B (TrkB) and p75 NT receptor (p75NTR). matBDNF binding to TrkB induces the activation of several signaling pathways, leading to cellular differentiation, survival and synaptic plasticity, among others. On the other hand, proBDNF can be secreted and it can mediate apoptosis through its interaction with a dual receptor system composed of p75NTR and the type I transmembrane protein sortilin (Teng et al., 2005, 2010). When p75NTR is activated by matBDNF or proBDNF, the main signal produces hippocampal long-term depression and neuronal death (Teng et al., 2005; Woo et al., 2005; Sun et al., 2012). However, when it is expressed together with TrkB, p75NTR may reduce TrkB ubiquitination and thus prolong TrkB signaling (Makkerh et al., 2005). It has been described that during hippocampal neuron development, low concentrations of endogenously-produced matBDNF or NT-4 protect TrkB-expressing neurons from p75NTR-mediated apoptosis (Friedman, 2000). This supports the notion that the lack of TrkB signaling makes neurons more vulnerable to NT-induced cell death.

Several studies have shown that seizures induce changes in the expression of NTs, proneurotrophins (pro-NTs) and their receptors (Unsain et al., 2008; Friedman, 2010; VonDran et al., 2014; Greenwood et al., 2018). Pilocarpine administration to rats, previously treated with lithium, leads to a prolonged seizure condition called *status epilepticus* (SE), which in turn results in cell death in several areas of the brain (Fujikawa, 1996). SE stimulates matBDNF and proBDNF protein expression (Rudge et al., 1998; Unsain et al., 2008); and matBDNF overproduction and release leads to a rapid downregulation of TrkB (Unsain et al., 2009). The role of endogenous matBDNF and proBDNF in SE-induced neuronal damage has been previously reported (Unsain et al., 2009). However, the strategies used in that study were not useful to differentiate the role of each form of BDNF in neuronal death after SE.

In this study, we used an *in vitro* and an *in vivo* model of SE to determine the specific role of both NT in the cell death process. We found that the neuronal death induced by SE is exacerbated in the absence of TrkB signaling.

## Materials and Methods

### Animal Housing

Adult male 2–2$\frac{1}{2}$-month-old Wistar rats (250–350 g) were used (Instituto Ferreyra, Córdoba, Argentina). The animals were housed at a constant temperature, with a 12 h-light/dark cycle, and had *ad libitum* access to food and water. The USA National Research Council Guide for the Care and Use of Laboratory Animals Guidelines (National Research Council, 1996) were followed. The animal model protocol used for this research was approved by the IIByT Animal Care and Use Committee, Resolution #1/2015. Care was taken to minimize the number of animals used per experiment as well as their suffering.

### Culture of Hippocampal Neurons

Primary cultures of embryonic day18 (E18) rat hippocampal neurons were prepared as described by Kaech and Banker (2006), with minor modifications. In brief, both hippocampi from each animal were mechanically dissociated and plated in a maintenance medium containing Neurobasal medium (NB) plus B27 supplement (Gibco), GlutaMax supplement (Gibco) and 0.5% Penicillin/Streptomycin (Gibco) at a density of 35,000 cells/cm^2^, in 35 mm plastic dishes coated with 0.1 mg/ml poly-L-lysine. The second day after plating, 10 μM Ara C was added to the culture to avoid growth of glial cells. Cultures were maintained 10–12 DIV at 37°C in a 95% air/5% CO_2_ atmosphere, and were fed twice a week with maintenance medium.

### Hippocampal Neurons-Astrocytes Co-cultures

Cultures from hippocampal astrocytes were obtained from P0 to P2 rats. Hippocampi were dissected out from the brains, cut into small sections, mechanically dissociated using a fire-polished Pasteur pipette, and plated in 25 cm^2^ poly-D-lysine-coated flasks with growth medium containing DMEM + 10% FBS + 0.5% Penicillin/Streptomycin. The glial mixed culture was grown to confluence (7–10 DIV) and non-astrocyte cells were removed by shaking the bottles containing the culture in an orbital shaker at 37°C (Mccarthy and de Vellis, 1980; Cragnolini et al., 2018). Then, astrocytes were trypsinized, plated onto glass coverslips, and cultured for 6–8 days in growth medium. Once astrocytes reached confluency again, hippocampal embryonic neurons from E18, obtained as described previously, were plated onto the astrocyte monolayer in maintenance medium (NB medium containing B27 supplement, glutaMAX, and 0.5% Pen/Strep). Fifty percent of the medium was replaced by fresh medium after 24 h and every 48 h.

### *In vitro* Model of Status Epilepticus

SE was induced in pure hippocampal neuronal cultures or neuron-astrocyte co-cultures after 10–12 DIV. The SE model was previously described by Sombati and DeLorenzo (1995) and consisted of exposing the cell culture to a MgCl_2_-free buffer solution which induces a continuous epileptiform high-frequency-bursts discharges. Briefly, growth medium was replaced by a buffer containing 145 mM NaCl, 2.5 mM KCl, 10 mM HEPES, 2 mM CaCl_2_, 10 mM glucose, 0.002 mM glycine, with or without 1 mM MgCl_2_ (control and SE, respectively), pH 7.3. SE was induced during 3 h, after which, medium was changed to maintenance medium to stop the insult. matBDNF scavenger, TrkB-Fc (0.7 μg/mL, R&D Systems) was added to the cell culture immediately after SE termination, as indicated in the results section. The concentration of TrkB-Fc was chosen based on previous reports (Yang et al., 2009).

### Surgery and Intrahippocampal Infusion Procedure

Twenty-two-gauge guide cannulas were implanted in the dorsal CA1 region of the left hippocampus of male Wistar rats anesthetized with ketamine-xylazine (100:15 mg/kg). Implantation was performed at the following Paxinos and Watson (2007) stereotaxic coordinates −3.2 anterior, +2.7 lateral, 1.2 ventral. A screw was placed in the skull near the cannulas to secure them, and dental acrylic was used to fix both screw and cannulas to the skull. After the surgery, animals were let to recover for 4–5 days before being induced to SE prior to infusion.

### *In vivo* Induction of Status Epilepticus

SE was induced by an intraperitoneal injection of 3 meq/kg lithium chloride and 12–16 h later was follow by an injection of 40 mg/kg pilocarpine hydrochloride (Sigma, St. Louis, MO, USA). Two hours after the onset of SE, 10 mg/kg diazepam (Hoffmann-La Roche Ltd, Basel, Switzerland) was injected intraperitoneally to stop seizure activity. To determine the onset of SE, animals were closely monitored, and seizure activity was assessed based on the previously described correlation between electrophysiological measures and observed seizures (Tremblay et al., 1984; Unsain et al., 2008). Experimentally, the control group consisted of animals that received saline solution instead of pilocarpine.

*In vivo* infusions were performed unilaterally into the hippocampal CA1 immediately after seizure termination. A Hamilton precision pump was used to administer the reagents at a rate of 1 μL/min and connected to a 30G infusing cannula which passed through the end of the guide cannula by 1.8 mm, so the final ventral coordinate of the infusion was 3 mm. Two microliters of 1 μg/μl recombinant human TrkB-Fc chimera protein (R&D Systems, cat#688-TK-100. Minneapolis, MN, USA) were infused according to Vaynman et al. (2004), or 0.8 μg/μl anti-proBDNF antibody (cat# ANT-006; Alomone Labs. Jerusalem, Israel) was administered according to Unsain et al. (2009). When the desired volume was reached, the infusion cannula was left *in situ* for 2 min to allow the liquid to diffuse in the tissue. The precision of infusion placement was examined by Cresyl Violet Staining (Nissl staining) in serial histological sections as described in Unsain et al. (2009). Only experimental data obtained from animals with a correctly placed cannula were considered for analysis. At least 3–4 animals per experimental condition were included for the analysis.

### Determination of Neuronal Damage

*In vitro* neuronal damage was evaluated by immunostaining of the nuclear protein NeuN (NeuN, 1:1,000; cat# ABN78; Millipore), since the loss of NeuN labeling is explained by neuronal death (Liu et al., 2013; Gusel’nikova and Korzhevskiy, 2015; Danelon et al., 2016), which was detected by immunostaining. In addition, beta III tubulin (1:5,000; cat# 4466; Cell Signaling) was used as a marker of cellular integrity, while DAPI (1:10,000; cat# D9542; Sigma Aldrich, St. Louis, MO, USA) was used as a nuclear marker. Six random fields per coverslip were analyzed, and the percentage of neuronal death was calculated as the number of NeuN-positive neurons relative to the control. Each experiment was repeated three times with three replicates per experiment.

Neuronal damage *in vivo* was determined 24 h after SE termination. Animals were deeply anesthetized with ketamine/xylazine and perfused transcardially with physiological solution (0.8% NaCl, 0.4% glucose and 0.8% sucrose) followed by 4% paraformaldehyde (PFA; Sigma Aldrich, St. Louis, MO, USA) in phosphate buffered saline (PBS) solution. After perfusion, brains were post-fixed in 4% PFA for 1 h, then cryoprotected in 30% sucrose at 4°C for 3 days before their sectioning. Neuronal degeneration after pilocarpine-induced SE *in vivo* was assessed by Fluoro-Jade B (FJB) staining (Sigma Aldrich, St. Louis, MO, USA) as previously reported (Schmued et al., 1997; Schmued and Hopkins, 2000).

Images of sliced brains were digitally captured with a Nikon DS-5M color video camera connected to a Nikon Eclipse TE2000-U (Tokyo, Japan) inverted microscope. In each brain section, square fields were analyzed in the hilus (neurons within the polymorphic layer of the dentate gyrus), and in the CA1 (cells within the *stratum piramidale*). The degree of injury in the hippocampus of the ipsilateral hemisphere was quantitatively compared to the degree observed in the hippocampus of the contralateral (control) hemisphere within each section. *In vivo*, the percentage of FJB-positive neurons was calculated according to Unsain et al. (2009). In short, 6–8 consecutive sections surrounding the infusion site (ranging from −2.45 to −3.95 mm from Bregma) were analyzed to calculate ipsilateral neuronal damage in relation to contralateral damage. To facilitate the comparative analysis with data from animals with a variable extent of neuronal injury—a common feature of SE-induced damage—the extent of injury was expressed as the mean percentage of the sections analyzed per hemisphere.

Nissl staining of brain sections was also performed as previously described (Unsain et al., 2009). Briefly, 50 μm-thick hippocampal histological sections were mounted on gelatin-coated slides using an alcohol-gelatin solution. The slides were subsequently dried at 37°C overnight, defatted in xylene, re-hydrated, and stained with 0.5% Cresyl Violet acetate for 2 min at room temperature. Then, the slides were dehydrated in 70% alcohol, cleared in xylene, and coverslipped with DPX mounting medium (Sigma Aldrich, St. Louis, MO, USA).

### Western Blot

Both SE-induced and control animals were sacrificed by decapitation 24 h after diazepam administration. The left and right dorsal hippocampi were removed as described in Unsain et al. (2009) and homogenized separately in RIPA buffer (50 mM Tris–HCl pH 7.4, 150 mM NaCl, 1% NP-40, 1% Triton X-100, 10% glycerol) containing protease and phosphatase inhibitors (Sigma Aldrich, St. Louis, MO, USA). Homogenates were cleared of debris twice by centrifugation at 5,000 *g* for 7 min. Protein concentration in the supernatant was determined by Bradford (1976). Protein samples were boiled in gel-loading buffer and separated by SDS-PAGE (10% for TrkB and pTrkB analyses, and 15% for matBDNF and proBDNF determination). Western blot was performed using nitrocellulose membranes (Bio-Rad Laboratories Inc., PA, USA), which were incubated in 5% non-fat milk/0.05% Tween/Tris-buffer for 2 h at room temperature, followed by overnight incubation at 4°C with the primary target antibody. Rabbit polyclonal anti-TrkB (1:500; cat# sc:8316. Santa Cruz Biotechnology, Dallas, TX, USA), rabbit monoclonal anti-phosphoTrkB^Y817^ (1:500; cat# 2149-1. Abcam), rabbit polyclonal anti-BDNF (1:500; cat# sc:546. Santa Cruz Biotechnology, Dallas, TX, USA), or rabbit polyclonal anti-proBDNF (1:1,000; cat# ANT-006. Alomone Labs) were used as primary antibodies and anti-rabbit HRP conjugated was used as secondary antibody (1:1,000; cat# 715-035-150. Jackson ImmunoResearch). Anti-proBDNF does not cross react with mature BDNF as determined by the manufacturer’s specifications and by our own analysis (Unsain et al., 2009). Protein band detection was performed by ECL followed by exposure to Kodak X-OMAT films (Kodak, Rochester, NY, USA). Protein loading control was determined by re-probing the membranes with a monoclonal anti-βIII tubulin antibody (1:10,000; cat# T8660. Sigma, St. Louis, MO, USA) followed by incubation with a secondary antibody anti-mouse HRP conjugated (1:1,000; cat# 715-035-150. Jackson ImmunoResearch). Bands were analyzed using the gel-scanning integrated optical density software ImageJ 1.44p (NIH, Bethesda, MD, USA). Protein level was determined from at least three animals per experimental group.

### Co-immunoprecipitation

After SE termination, the left and right hippocampus were obtained and homogenized in modified RIPA buffer plus protease and phosphatase inhibitors. Homogenates were centrifuged at 14,000 rpm for 10 min. Five-hundred micrograms of total protein from each supernatant were pre-cleared 3 h at 4°C with 10 μl Protein A/G PLUS-agarose (Santa Cruz Biotechnology, Dallas, TX, USA). After centrifugation, the pre-cleared supernatants were incubated with 2 μg anti-proBDNF antibody (cat# ANT-006; Alomone Labs) or 2 μg TrkB-Fc at 4°C overnight. Then, 12 μl of Protein A/G PLUS-Agarose was added. Immunoprecipitates were washed three times with ice-cold modified RIPA buffer before elution in SDS sample buffer. Samples were analyzed by Western blot using rabbit anti-p75NTR primary antibody (1:500; cat# sc-6188; Santa Cruz Biotechnology, Dallas, TX, USA), rabbit anti-TrkB primary antibody (1:500; cat# sc:8316; Santa Cruz Biotechnology, Dallas, TX, USA), rabbit anti-BDNF (1:1,000, sc:546; Santa Cruz) or rabbit anti-proBDNF (1:1,000, ANT-006; Alomone Labs). Membranes were incubated with a rabbit secondary antibody before incubation with the primary rabbit antibody, as IgG loading control.

### Statistical Analysis

Quantitative data are presented as mean ± SEM. The relative protein level between groups was analyzed by One-way ANOVA or Two-way ANOVA followed by Tukey *post hoc* test. For quantification of NeuN-positive cells and FJB-positive cells, a Nested ANOVA test was used. *p* values < 0.05 were considered statistically significant.

## Results

### matBDNF Prevents Neuronal Death Induced by proBDNF

**Figure 1:**
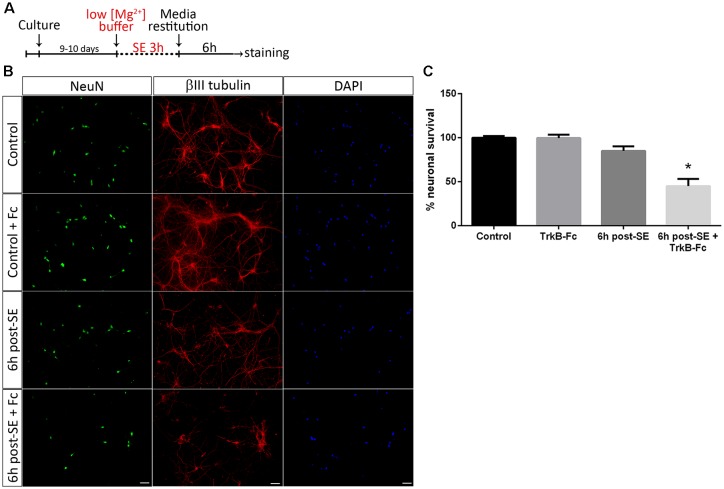
Sequestering brain-derived neurotrophic factor (BDNF) induces neuronal death after status epilepticus (SE) *in vitro*. **(A)** SE was induced in neuronal cultures for 3 h (“No Mg^2+^ buffer”) followed by growth media restitution. Controls cultures received a buffer with MgCl_2_. After 6 h, cells were immunostained for NeuN and βIII-tubulin, plus DAPI. **(B)** Representative micrographs showing *in vitro* hippocampal neurons stained with NeuN (green), DAPI (blue) and βIII-tubulin (red) in control hippocampal neurons and 6 h post-SE, with or without the infusion of tropomyosin receptor kinase B-Fc (TrkB-Fc). Scale bar = 50 μm. **(C)** Quantification of NeuN-positive cells. Data are expressed as mean ± SEM (*n* = 3 independent experiments with three pseudo-replicates per experiment). Asterisk indicates *p* < 0.001 compared to control by one-way ANOVA followed by Tukey’s *post hoc* analysis.

To unravel the mechanisms underlying neuronal death after SE, a well-known *in vitro* model of *SE* (Sombati and DeLorenzo, 1995) was used. We have recently shown that SE induces death in a pure culture of hippocampal neurons 12 h after SE termination. However, in 6 h, post-SE signs of neurodegeneration such as spheroids formation and decrease in the length of neurites can be observed (Danelon et al., 2016). Taking into account these results, we investigated the specific roles of matBDNF and proBDNF in neuronal death after SE. Cell death induced by SE in a culture of highly-pure hippocampal neurons (Supplementary Figure S1) was assessed by quantifying the presence/absence of NeuN protein. The addition of TrkB-Fc, a matBDNF scavenger (Shelton et al., 1995) to the neuronal culture immediately after SE, induced a remarkable 50% cell death 6 h post-insult, a time point at which cell death had not been previously detected (Figures 1A–C; one-way ANOVA *p = 0.001). Considering that an *in vitro* proBDNF/p75NTR interaction increase 6 h post-SE (Danelon et al., 2016) was also demonstrated, these results suggest that, in absence of matBDNF, proBDNF/p75NTR signaling would predominate and increase neuronal death.

Since the discovery of the capacity of proBDNF to mediate several biological functions, attempts have been made to determine whether proBDNF can bind to TrkB receptors, with contradictory results (Mowla et al., 2001; Fahnestock et al., 2004; Fayard et al., 2005; Teng et al., 2005; Boutilier et al., 2008; Koshimizu et al., 2010; Gaub et al., 2016). However, many studies had demonstrated that proBDNF exerts its proapoptotic effects by binding to the p75NTR-sortilin complex (Beattie et al., 2002; Harrington et al., 2004; Taylor et al., 2012). In this study, TrkB-Fc was used to differentiate the effects of matBDNF and proBDNF separately. To rule out the possibility that proBDNF was able to bind to TrkB, a co-immunoprecipitation assay was performed. Protein extracts from neuronal cultures that could have undergone SE or not were immunoprecipitated with an antibody against proBDNF, and subsequently analyzed by Western blot with an anti-TrkB antibody. An interaction of TrkB with proBDNF was not detected, neither in control nor in SE samples (Supplementary Figure S2A), suggesting that proBDNF does not bind to TrkB. In addition, to confirm that TrkB-Fc does not interact with proBDNF, a co-immunoprecipitation was performed, using samples from control and SE hippocampus. The homogenates were mixed with TrkB-Fc that was immunoprecipitated and then Western blots were probed for matBDNF and proBDNF. An interaction of TrkB-Fc with matBDNF was observed, but not with proBDNF (Supplementary Figures S2B,C). Together, these results confirm that TrkB-Fc specifically scavenges matBDNF and not proBDNF.

**Figure 2:**
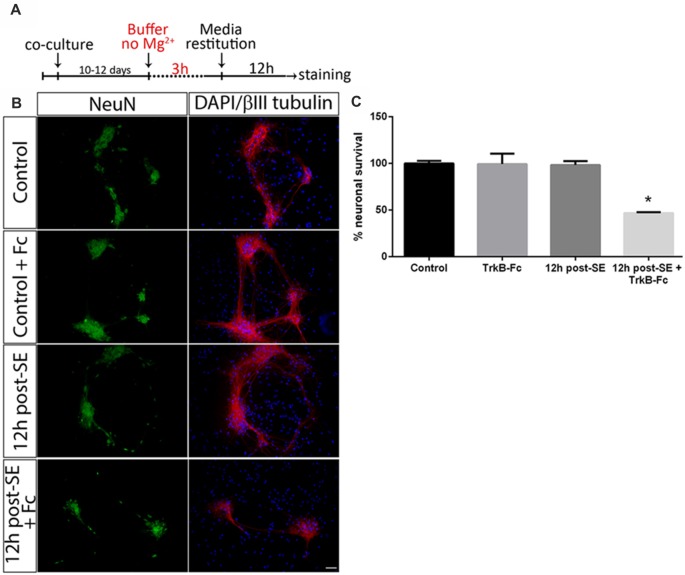
Sequestering BDNF induces neuronal death after SE in neurons-astrocytes co-cultures. **(A)** SE was induced in co-cultures for 3 h (“No Mg^2+^ buffer”) followed by growth media restitution. Control cultures received a buffer with MgCl_2_. After 12 h, cells were immunostained for NeuN and βIII-tubulin, plus DAPI. **(B)** Representative micrographs of NeuN (green), βIII-tubulin (red) and DAPI (blue) staining in control, control + TrkB-Fc (Fc), or 12 h post-SE 12 h post-SE + TrkB-Fc hippocampal neurons. Scale bar = 50 μm. **(C)** Quantification of NeuN-positive cells in co-cultures. Data are expressed as mean ± SEM (*n* = 3 independent experiments with three pseudo-replicates per experiment). Asterisk indicates *p* < 0.05 compared to control cultures by one-way ANOVA followed by Tukey’s *post hoc* analysis.

Astrocytes regulate several neuronal functions and provide proBDNF clearing and recycling in order to release BDNF (Vignoli et al., 2016). It was evaluated whether the presence of astrocytes may prevent neuronal death after SE induced by proBDNF. As previously reported, death of hippocampal neuronal cells in a highly-pure culture increased 12 h post-SE (Danelon et al., 2016); however, no significant neuronal death was found in presence of astrocytes at the same time point. When TrkB-Fc was added immediately after SE, neuronal death significantly increased 12 h after the insult (Figures 2A–C; one-way ANOVA *p < 0.0001).

In summary, in both *in vitro* cultures (pure hippocampal culture and co-culture with astrocytes), sequestering of matBDNF induces an increase in post-SE neuronal death. This phenomenon could be explained due to an imbalance between matBDNF/TrkB signaling and proBDNF/p75NTR signaling.

**Figure 3:**
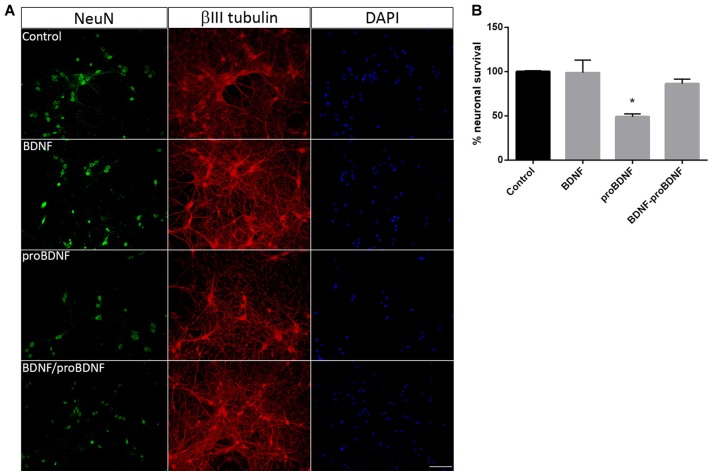
BDNF prevents proBDNF-induced neuronal death. **(A)** Representative micrographs of hippocampal neurons stained with NeuN, βIII-tubulin and DAPI. The following conditions were studied: untreated control, cells treated with 100 ng/ml BDNF, with 100 ng/ml proBDNF, or with BDNF + proBDNF. Scale bar = 50 μm. **(B)** Quantification of NeuN-positive cells. Data are expressed as mean ± SEM (*n* = 3 independent experiments with three pseudo-replicates per experiment). Asterisk indicates *p* = 0.024 compared to control by one-way ANOVA followed by Tukey’s *post hoc* analysis.

In order to confirm the role of proBDNF in SE-induced neuronal death, pure hippocampal neurons were incubated with exogenous proBDNF, matBDNF or both at the same time and the neuronal death after 24 h was determined. Although we do not rule out the possibility that these NT are released by neurons under basal conditions, several studies had demonstrated that there is no secretion of matBDNF or proBDNF unless they receive a depolarizing stimulus (Goodman et al., 1996; Gärtner and Staiger, 2002; Nagappan et al., 2009; Yang et al., 2009). *In vitro* treatment of hippocampal neurons with 100 ng/ml proBDNF reduced neuronal survival by approximately 50%, shown by a decrease in the NeuN staining in neurons in Figure 3A. Also, βIII tubulin staining showed spheroids formation along the neurites in proBDNF treated neurons, an event related to neuronal degeneration. The addition of matBDNF (100 ng/ml) blocked proBDNF-induced cell death (Figures 3A,B one-way ANOVA *p = 0.024), supporting the idea that TrkB signaling is necessary to prevent proBDNF-induced neuronal death.

### Absence of matBDNF Increases Neuronal Death After SE *in vivo*

**Figure 4:**
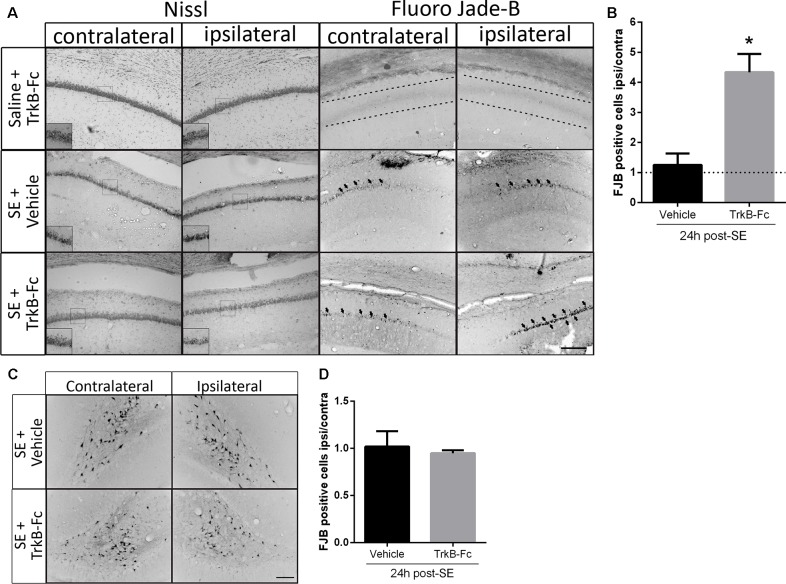
TrkB-Fc increases neuronal death after SE *in vivo*. **(A)** Micrographs of hippocampal CA1 stained with Nissl (left panels) or with Fluoro-Jade B (FJB; right panels) show representative brain sections of animals with each treatment. Insets show, at higher magnification, morphological alterations evidenced by Nissl staining. Either TrkB-Fc or vehicle were infused in the hippocampi of one hemisphere (ipsilateral) of control (saline) and SE animals. **(B)** Quantification of the ipsilateral/contralateral FJB positive neurons in CA1 area. **(C)** Micrographs of hippocampal hilus stained with FJB show representative brain sections of animals with each treatment. **(D)** Quantification of the ipsilateral/contralateral FJB positive neurons in hippocampal hilus. Results are expressed as a mean ± SEM of three animals per treatment. Asterisk indicates *p* < 0.05 compared to vehicle by nested ANOVA. Scale bar = 200 μm.

We have previously reported that *in vivo* infusion of a matBDNF-blocking antibody immediately after the SE decreases post-SE neuronal death (Unsain et al., 2009); however, the antibody used recognizes matBDNF as well as proBDNF. Thus, to identify the role of each form of BDNF in neuronal death resulting from SE *in vivo*, animals were unilaterally infused with TrkB-Fc into the hippocampal CA1 immediately after seizure termination. Twenty-four hours later, neuron cell survival was evaluated by Nissl staining and by FJB staining. We found that degenerating neurons (FJB positive cells) increased in the hippocampal CA1 at a similar level in both hemispheres in animals infused with vehicle. No neuronal damage was induced by TrkB-Fc alone in control animals (animals that received saline instead of pilocarpine). On the contrary, a four-fold increase in neuronal death was shown in response to post-SE infusion of TrkB-Fc compared to the contralateral vehicle-infused hippocampus (Figures 4A,B; unpaired *t*-test *p = 0.0130). The effect of the infusion of TrkB-Fc after SE on neuronal death did not extent to the hilus of the dentate gyrus since the number of degenerating neurons was similar in the hilus of both hippocampi (Figures 4C,D). We performed Nissl staining as another assessment of the damage in the hippocampus after SE and TrkB-Fc infusion. As it can be noticed in Figure 4A, left panels, Nissl staining yielded to similar results to those obtained by FJB.

### TrkB Downregulation Facilitates Neuronal Death After SE *in vivo*

**Figure 5:**
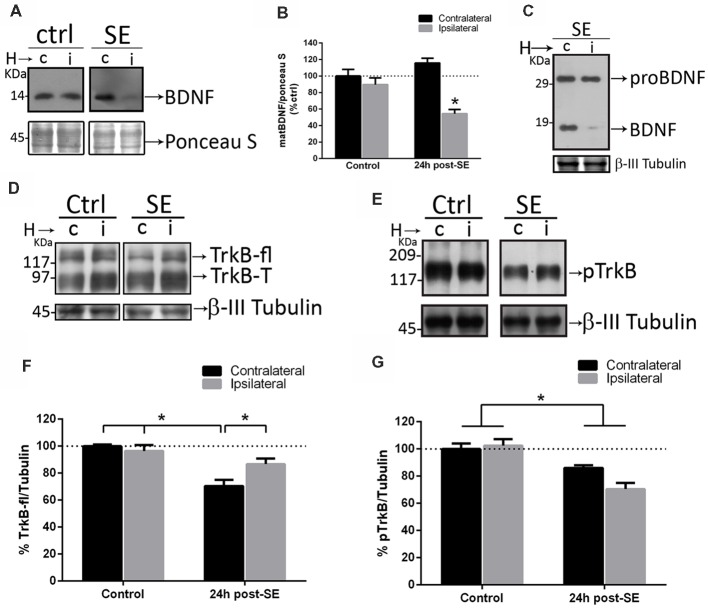
Sequestering BDNF prevents TrkB signaling. TrkB-Fc was infused in the hippocampi of one hemisphere (ipsilateral, i) of control and SE animals. The dorsal hippocampus from non-infused side (c) and infused side (i) were homogenized separately and analyzed by western blot to determine BDNF **(A)**, proBDNF **(C)**, TrkB **(D)** and pTrkB **(E)**. Panels **(B,F,G)** show the quantifications for BDNF **(B)**, TrkB **(F)** and pTrkB **(G)**. Black bars represent non-infused hippocampus and gray bars TrkB-Fc infused sides. Results are expressed as mean ± SEM of four animals per treatment. Asterisks indicate *p* < 0.05 by two-way ANOVA followed by Tukey’s *post hoc* analysis.

Since sequestering extracellular matBDNF *in vivo* increases neuronal death after SE, the hypothesis is that, upon depletion of matBDNF/TrkB signaling, proBDNF/p75NTR signaling predominate inducing neuronal death. SE and control animals were unilaterally infused with TrkB-Fc into the hippocampal CA1 area. Twenty-four hours later, septal halves from both hippocampi were obtained separately (Unsain et al., 2009), and matBDNF and proBDNF protein levels were determined by Western blot. We found that SE decreased matBDNF protein in the ipsilateral TrkB-Fc-infused hippocampus (Figures 5A,B), however, in the same animals, proBDNF levels were not affected by the infusion of TrkB-Fc (Figure 5C). As mentioned before, SE induces a 30% decrease in the levels of the TrkB full length (TrkB-fl) in the control hippocampus (Unsain et al., 2008) which was prevented by TrkB-Fc infusion (Figures 5D,F; two-way ANOVA *p = 0.01). These results support the idea that the marked increase in matBDNF that occurs after SE results in TrkB-fl downregulation.

TrkB-fl is activated by phosphorylation after matBDNF binding. Then, the levels of phosphorylated TrkB (p-TrkB) were determined in the rat hippocampus after SE in control and TrkB-Fc infused animals. We found a 30% decrease in pTrkB levels, in both hemispheres after SE (Figures 5E,G; two-way ANOVA *p = 0.0017), indicating that TrkB signaling is attenuated in the injured hippocampus.

**Figure 6:**
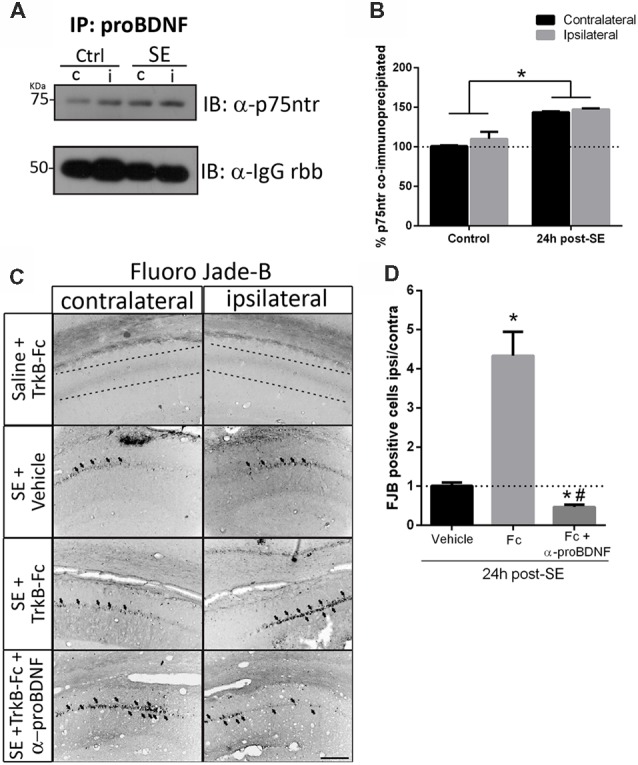
Blockage of proBDNF prevents neuronal death even when BDNF is sequestered.** (A)** Representative immunoblot showing p75NTR co-immunoprecipitated with proBDNF in the non-infused side (c) and infused side (i) in control and SE animals, and quantification **(B)**. Mean ± SEM are indicated. Protein levels were quantified in three animals per group. Asterisks indicate *p* < 0.05 compared to control contralateral by two-way ANOVA followed by Tukey’s *post hoc* analysis. **(C)** Micrographs of hippocampal CA1 stained with FJB show representative animals from each treatment, and quantification **(D)**. Results are expressed as mean and SEM of three to five animals per treatment. Asterisks indicate *p* < 0.05 compared to vehicle by nested ANOVA followed by Tuckey’s *post hoc* analysis. Hashtag indicate *p* < 0.05 compared to TrkB-Fc by nested ANOVA followed by Tukey’s *post hoc* analysis. Scale bar = 200 μm.

Figures 5A,C show that, when matBDNF was sequestered, proBDNF levels were not modified. Thus, this pro-NT could still be able to bind to p75NTR. Therefore, the predominant signal would be the induction of neuronal death *via* proBDNF/p75NTR. To test this hypothesis, we evaluated whether the interaction between proBDNF and p75NTR was modified after SE in the absence of matBDNF. As shown in Figure 6, while the interaction between proBDNF and p75NTR was not affected by the infusion of TrkB-Fc, it was increased by ~47% in response to SE (Figures 6A,B; two-way ANOVA *p < 0.0001).

Considering these results, it was then determined whether proBDNF potentiates the SE-induced neuronal death in the absence of matBDNF. TrkB-Fc was infused *in vivo* together with an antibody that only recognizes proBDNF (anti-proBDNF) immediately after seizures. Twenty-four hours later, neuronal survival was analyzed in ipsilateral and contralateral hippocampi by Fluro-Jade B. We found that, not only the administration of anti-proBDNF abolished TrkB-Fc-induced neuronal death, but it also reduced the level of neuronal death triggered by SE (Figures 6C,D one-way ANOVA *p < 0.0001), suggesting that proBDNF plays an important role in mediating neurodegeneration after SE.

## Discussion

NT elicit a paradoxical effect on neurons, since they can induce either neuronal survival or death, depending on which receptor and signaling pathways are activated. Our previous work revealed an intricate regulation of matBDNF and proBDNF in excitotoxicity that also involved the downregulation of TrkB and the upregulation of p75NTR (Unsain et al., 2008, 2009). Therefore, it was important to establish the relative contribution of each form of BDNF to the mechanisms involved in neuronal survival and death. In this work we demonstrated, *in vitro* and *in vivo*, that proBDNF signaling through p75NTR exacerbated the SE-induced neuronal death in absence of matBDNF/TrkB signaling.

matBDNF is known to promote neuronal survival, differentiation, and synaptic plasticity, although in intact mature neurons the endogenous matBDNF is not needed for survival (Shulga et al., 2012) and, under this condition, its expression is low (Goodman et al., 1996; Gärtner and Staiger, 2002; Nagappan et al., 2009; Yang et al., 2009). Our results and those from other groups agree that seizures increase the expression of matBDNF NT in several brain areas (Ernfors et al., 1991; Bengzon et al., 1993; Larmet et al., 1995; Rudge et al., 1998; Tandon et al., 1999; Lähteinen et al., 2002; Xu et al., 2004; Unsain et al., 2008, 2009). However, the reasons and consequences of this shift are not well known. Endogenous matBDNF have been associated to a possible neuroprotective effect (Tandon et al., 1999), but the exogenous administration has been reported to attenuate the SE progression (Larmet et al., 1995) or cause no effect on neuron survival (Rudge et al., 1998).

Neurons may also secrete proBDNF, the immature form of BDNF and our results, together with those from other groups, demonstrate that proBDNF levels increase under pathological conditions such as SE (Unsain et al., 2008; VonDran et al., 2014; Thomas et al., 2016). Whether or not proBDNF is able to bind to TrkB activating signaling cascades that lead to neuronal survival was analyzed in many studies, but the results are still a matter of discussion. Our results suggest that the endogenous proBDNF expressed in hippocampus would not be able to bind to TrkB under physiological conditions, nor after an excitotoxic event. This result is in agreement with studies that showed that proBDNF is ineffective in activating TrkB (Teng et al., 2005; Koshimizu et al., 2010; Gaub et al., 2016), indicating that proBDNF signals through p75NTR.

We have previously shown that matBDNF and proBDNF facilitate neuronal death after SE (Unsain et al., 2009); however, the methodology used did not allow to distinguish between matBDNF and proBDNF actions. In this study, matBDNF was sequestered *via* TrkB-Fc to demonstrate the role of proBDNF in neurodegeneration. TrkB-Fc is a BDNF scavenger unable to enter the cells, thus it will solely sequester the extracellular matBDNF. The intracerebral infusion of TrkB-Fc had been previously used to inhibit the development of kindling and it was probed to inhibit TrkB activation (Binder et al., 1999). We showed in both models of SE, *in vitro* and *in vivo*, that the scavenging of matBDNF post-SE increases neuronal death. In addition, we demonstrated that overproduction of matBDNF after SE is the sole trigger of post-SE TrkB downregulation, since withdrawing this NT restores TrkB level in the hippocampus.

It has been demonstrated that the constitutive release of matBDNF and proBDNF to the extracellular space is low in basal conditions (Goodman et al., 1996; Gärtner and Staiger, 2002; Nagappan et al., 2009; Yang et al., 2009), however, the release of both proteins increase after SE (Nawa et al., 1995; Schmidt-Kastner et al., 1996; Rudge et al., 1998). Taking this into account, we hypothesize that after SE, the matBDNF as well as proBDNF start to be released to the extracellular space. In the presence of TrkB-Fc, only the matBDNF is sequestered. Then, the matBDNF bound to TrkB-Fc is cleared out from the extracellular space and, for that reason, we observed a decrease in matBDNF levels. This idea is in agreement with our results and those obtained by other groups that demonstrate the clearing of other molecules such as antibodies and antisense oligonucleotides 24 h after being infused to the hippocampus (Bekinschtein et al., 2007; Unsain et al., 2009; Anastasía et al., 2011). When BDNF is absent, it allows to proBDNF signaling to occur through p75NTR, and therefore to induce neuronal death.

ProNTs have been shown to contribute to neuronal death by signaling through p75NTR and sortilin receptors (Lee et al., 2001; Beattie et al., 2002; Nykjaer et al., 2004; Teng et al., 2005; Massa et al., 2006; Domeniconi et al., 2007; Koshimizu et al., 2010; Tauris et al., 2012; Taylor et al., 2012). It has been described that the inhibition of p75NTR signaling (Troy et al., 2002; Volosin et al., 2008; Song et al., 2010) or proNT cleavage (Le and Friedman, 2012; Thomas et al., 2016) prevents proNT upregulation and neuronal death after SE. Moreover, proNGF could bypass matBDNF induction of neuronal survival by increasing PTEN activity (Song et al., 2010). Furthermore, PTEN phosphatase activity could be modulated by SE, since both TrkB level and TrkB phosphorylation decrease before neuronal death (Unsain et al., 2008). Induction of proNGF and p75NTR has been observed in several injury models and neurodegenerative diseases (Harrington et al., 2004; Song et al., 2010; Alder et al., 2016) and the blockage of proNGF/p75NTR signaling is effective in limiting neuronal apoptosis (Fahnestock et al., 2001; Beattie et al., 2002; Harrington et al., 2004; Arnett et al., 2007; Jansen et al., 2007; Tep et al., 2013). In contrast, the role of endogenous proBDNF in neurodegenerative diseases has been less explored (Peng et al., 2005; Taylor et al., 2012), although it has been associated with neuronal death. Our results may reflect the importance of proBDNF and p75NTR signaling in mediating neuronal death over the course of a neurodegenerative condition such as SE.

Astrocytes play an important role during normal development as well as during an injury (Liddelow and Barres, 2017). It is well known that they react after SE (Vargas-Sánchez et al., 2018), although whether they have a protective role or not in SE is still questioned. In our previous work we used an astrocyte-free culture condition and observed that the SE *in vitro* caused a significant neuronal lost at 12 h post-SE (Danelon et al., 2016). Surprisingly, the SE-induced neurodegeneration was prevented when astrocytes were present in the culture. This result provide evidence that astrocytes play a key role in protecting neurons from SE-induced death *in vitro*, although the mechanisms involved in neuroprotection are still a matter of study. In relation to this result, it has been shown that proNGF and proBDNF expression (Volosin et al., 2006, 2008) are increased in astrocytes after seizures. In addition, astrocytes incorporate the excess release of proBNDF *via* p75NTR during excitotoxic insults and, at the same time, they may release several trophic factors (Bergami et al., 2008; Vignoli et al., 2016). The p75NTR expression also increases in astrocytes after SE (Cragnolini et al., 2009), which could serve as a receptor that eventually uptakes proBDNF and releases matBDNF as suggested by Bergami et al. (2008). We guess that the capabilities of astrocytes for uptaking proBDNF are limited since they did not protect neurons in the presence of TrkB-Fc, suggesting that the overwhelming proBDNF/p75NTR death signal predominates on neurons.

The SE severely damages the hippocampus causing neurodegeneration in several hippocampal subareas. The temporal sequence of neuronal death differs among them, and it is also affected by the model used to induce SE, even by the doses of pilocarpine used (Fujikawa, 1996; do Nascimento et al., 2012). In consistence with our previous work (Unsain et al., 2008, 2009), in this study we observed neuronal death in CA1 at 24 h after SE. The withdrawal of matBDNF produced a further increase in neuronal death in the CA1. This result supports the idea that SE induces a switch between TrkB and p75NTR receptors levels. In that context, proBDNF and/or matBDNF mainly interact with p75NTR leading to the neuronal death observed 24 h post-SE in the CA1 area. We showed that the administration of an antibody against proBNDF completely reverted the exacerbated death induced by SE plus TrkB-Fc. This result confirms that in the absence of matBDNF, proBDNF takes a preponderant role in inducing death. Indeed, similar results were obtained in other injury models, such as in an *in vivo* axotomy model in which neuronal death occurs after 1–3 days post-injury (Harrington et al., 2004; Shulga et al., 2012). In this model, matBDNF withdrawal by TrkB-Fc increased neuronal death, but when p75NTR was blocked, matBDNF deprivation did not induce death in axotomized neurons. Furthermore, plasmin decreases the number of apoptotic hippocampal neurons in TrkB-Fc treated (Shulga et al., 2012), suggesting that proBDNF and proNGF participate in neuronal death in the absence of TrkB-matBDNF signaling.

Contrarily to the effect observed in the CA1, further death in the hilus after the infusion of TrkB-Fc was not observed. The hippocampal hilus is more vulnerable to the SE than CA1 and, in fact, the neuronal death occurs immediately after SE (Fujikawa, 1996; Unsain et al., 2008, 2009). In our prior studies, we also reported a decrease in TrkB levels in CA1 that precedes the neuronal death observed 24 h post-SE, while in the hilus, the decrease in TrkB levels is concomitant with the neuronal injury. Although we did not discard the possibility that matBDNF/TrkB signaling is important for the neuronal survival after SE as it is in CA1, we suggest that the extension of the damage in the hilus after SE was so severe that did not support further neuronal death after scavenging matBDNF.

As described in Unsain et al. (2008) in the *in vivo* model of SE, the hippocampus is severely damaged, and in CA1, neuronal death occurs 24 h after seizures. In this study, we proposed that the exacerbated release of matBDNF after the SE insult could be a neuronal survival mechanism, since the matBDNF signal may serve to counteract the pro-apoptotic signals triggered by proBDNF/p75NTR. Our data shows that when the matBDNF/TrkB signaling decreases as a result of a matBDNF scavenger’s activity, the proBDNF/p75NTR becomes the predominant—pro-apoptotic—active pathway.

Our results strongly support the idea that the lack of balance in neurotrophic receptors under neurodegenerative conditions may determine the fate of neurons, and that, even in the presence of pro-NT, matBDNF/TrkB signaling plays a key role in neuronal survival.

## Author Contributions

All authors were responsible for the design of the study. LM and VD performed the experiments and analyzed the data. LM conducted statistical data analysis. VD and AC performed cell culture. The manuscript was drafted by LM. All authors critically reviewed the content and approved the final version for publication. DM supervised the project and conceived the original idea.

## Conflict of Interest Statement

The authors declare that the research was conducted in the absence of any commercial or financial relationships that could be construed as a potential conflict of interest.
