# Supplementary material for: Loss of TrkB Signaling Due to Status Epilepticus Induces a proBDNF-Dependent Cell Death
Source: Front Cell Neurosci. 2019 Feb 8;13:4. doi: 10.3389/fncel.2019.00004 (PMC6375841; doi:10.3389/fncel.2019.00004)
Supplement: Supplementary file 1 [file Data_Sheet_1.pdf]

Supplementary Material

Loss of TrkB signaling due to Status Epilepticus induces a proBDNF dependent cell death

Laura Ester Montroull[&], Víctor Danelon[&], Andrea Beatriz Cragnolini* and Daniel Hugo Mascó[*].

Laboratorio de Neurobiología, Instituto de Investigaciones Biológicas y Tecnológicas, Córdoba, Argentina.

[*] Correspondence:

Dr. Daniel H. Mascó

or

Andrea B. Cragnolini

Laboratorio de Neurobiología

Centro de Biología Celular y Molecular

Instituto de Investigaciones Biológicas y Tecnológicas (CONICET-FCEFyN, UNC)

Av. Vélez Sarsfield 1611, X5016 GCA, Córdoba, Argentina

dmasco@fcq.unc.edu.ar

anbecr@gmail.com

1. Supplementary Figures and Tables

1.1. Supplementary Figures

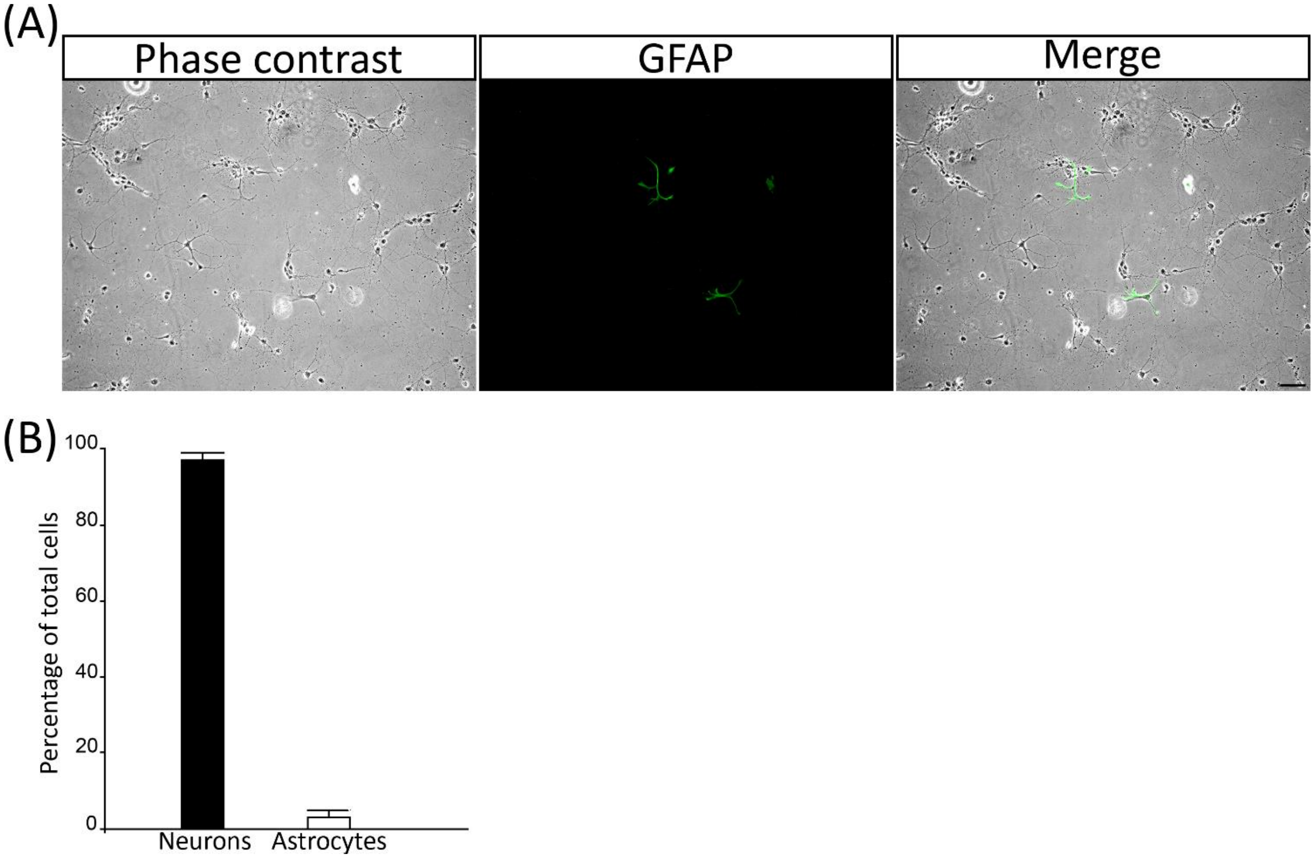

Supplementary Figure 1. (A) Representative micrographs showing hippocampal neurons cultures in phase contrast counterstained with astrocytic marker anti-GFAP. Scale bar: 50µm. (B) Quantification of the number of astrocytes present in neuronal cultures show scarce number of astroglial cells in neuronal cultures. Data are expressed as mean ± SEM (n=3 independent experiments with 3 pseudo-replicates per experiment).

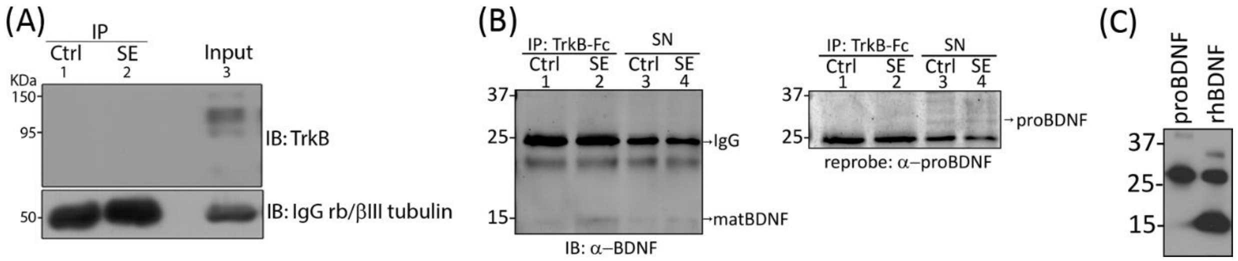

Supplementary Figure 2. TrkB is not able to interact with proBDNF. (A) Representative immunoblot showing not detection of proBDNF when TrkB is immunoprecipitated in control and 6h after SE *in vitro* (IP lanes: 1 and 2). Lane 3 shows TrkB immunoblot in the input. The membrane was probed against IgG rabbit to control for equal protein loading between wells and against βIII tubulin as a loading control for the input lane. (B) The hippocampus from control or SE animals were homogenized and immunoprecipitated with TrkB-Fc. Lines 1 and 2 from the left panel show matBDNF immunoprecipitated with TrkB-Fc. Lines 3 and 4 show also matBDNF in the supernatant. No interaction between TrkB-Fc and proBDNF was observed. Right panel show the same membrane re-probed with a specific antibody against proBDNF. Lines 3 and 4 show detection of proBDNF in the supernatant but not in the IP (line 1 and 2). (C) Positive controls for matBDNF and proBDNF were run to confirm the molecular weight of these proteins.
